# Dr. Blinn A. Harris

**Published:** 1917-02

**Authors:** 


**Dr. Blinn A. Harris,** N. Y. University 1885, of Norwich, Chenango Co., N. Y., died Dec. 23, as the result of serious cardiac disease of 5 years' duration.

